# Rothmund-Thomson syndrome and osteoma cutis in a patient previously diagnosed as COPS syndrome

**DOI:** 10.1007/s00431-016-2834-3

**Published:** 2016-12-30

**Authors:** M. C. van Rij, M. L. Grijsen, N. M. Appelman-Dijkstra, K. B. M. Hansson, C. A. L. Ruivenkamp, K. Mulder, R. van Doorn, A. P. Oranje, S. G. Kant

**Affiliations:** 1 Department of Clinical genetics, Leiden University Medical Centre, Postzone K5-R, PO box 9600, 2300 RC Leiden, The Netherlands; 2 Department of Dermatology, Leiden University Medical Centre, Leiden, The Netherlands; 3 Department of Endocrinology, Leiden University Medical Centre, Leiden, The Netherlands; 4 Kinderhuid.nl Teledermatology, Rotterdam, Dermicis Skin Clinic Alkmaar, Practice for Hair and skin, Breda, The Netherlands

**Keywords:** Rothmund-Thomson syndrome, Poikiloderma, *RECQL4* gene, Mental retardation/developmental delay/intellectual disability, Osteoporosis, Aneuploidy, Chromosomal instability, COPS syndrome, Osteoma cutis, Calcinosis cutis

## Abstract

We present a patient with poikiloderma, severe osteoporosis and a mild intellectual disability. At the age of 9 years, this patient was proposed to suffer from a novel disease entity designated as calcinosis cutis, osteoma cutis, poikiloderma and skeletal abnormalities (COPS) syndrome. At the age of 35, he was diagnosed with Hodgkin’s lymphoma. Recently, biallelic pathogenic variants in the *RECQL4* gene were detected (c.1048_1049delAG and c.1391-1G>A), confirming a diagnosis of Rothmund-Thomson syndrome (RTS). In the brother of this patient, who had a milder phenotype, a similar diagnosis was made.

*Conclusion*: We conclude that COPS syndrome never existed as a separate syndrome entity. Instead, osteoma cutis may be regarded as a novel feature of RTS, whereas mild intellectual disability and lymphoma may be underreported parts of the phenotype.

**Table:** 

**What is new:**
• Osteoma cutis was not a known feature in Rothmund-Thomson patients.
• Intellectual disability may be considered a rare feature in RTS; more study is needed.
**What is known:**
• RTS is a well-described syndrome caused by mutations in the *RECQL4* gene.
• Patients with RTS frequently show chromosomal abnormalities like, e.g. mosaic trisomy 8.

## Case description

A 34-year-old man with dysmorphic features, osteoporosis and recurrent fragility fractures with non-union was referred to the department of clinical genetics. At age 9, a clinical diagnosis of calcinosis cutis, osteoma cutis, poikiloderma and skeletal abnormalities (COPS syndrome) was made and reported in this journal [11]. In summary, he had dysmaturity (birth weight 2400 g at 40 weeks gestational age) and severe diarrhoea requiring parenteral feeding. At the age of 3, he suffered from meningitis due to mumps infection. Furthermore, at the age of 4, subcutaneous tumours, osteomas, with a maximal diameter of 3 cm were removed from the ankles, knees and forehead. Skeletal abnormalities were observed with hypoplastic patellae and delayed bone maturation. At the age of 15 years, a diagnosis of coeliac disease was made for which he was started on a diet and vitamin D supplementation. He suffered from multiple fragility fractures of both the tibiae, the right elbow, the left patella and the metatarsal bone V of his right foot, complicated by pseudo arthroses. A bone mass density measurement was performed at age 27, showing a T-score of −2.6 femur and 2.4 lumbar vertebrae, consistent with a diagnosis of osteoporosis. Treatment with alendronate was initiated and while on treatment his bone mass increased and no fractures occurred. Treatment was discontinued at the age of 30 and at age 34. He suffered from a mild intellectual disability for which he attended special educational programs.

**Fig. 1:**
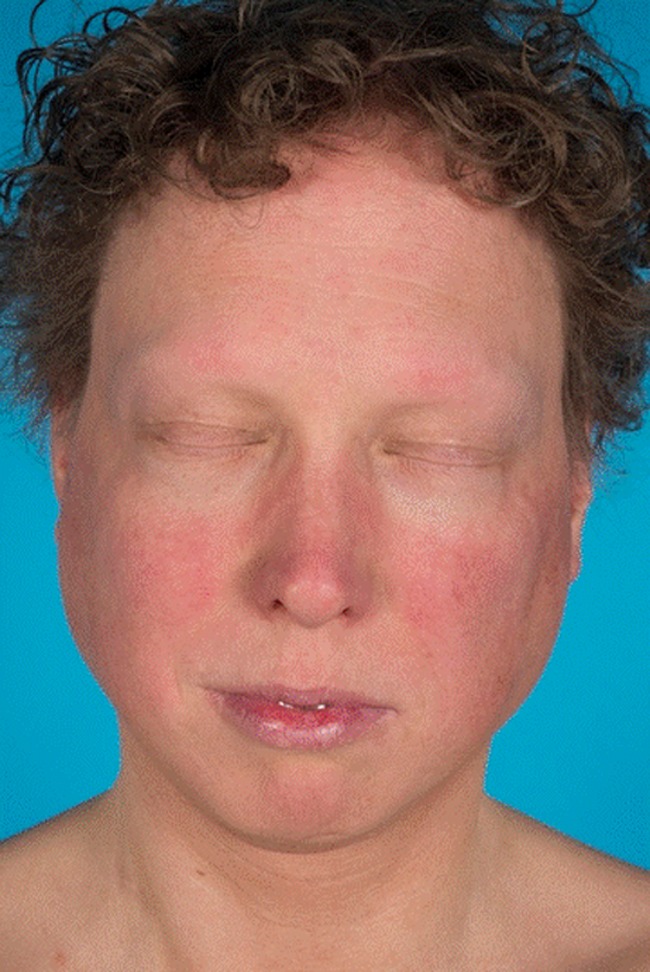
Facial features at age 34. Note: absent eye brows and eye lashes, small nose

**Fig. 2:**
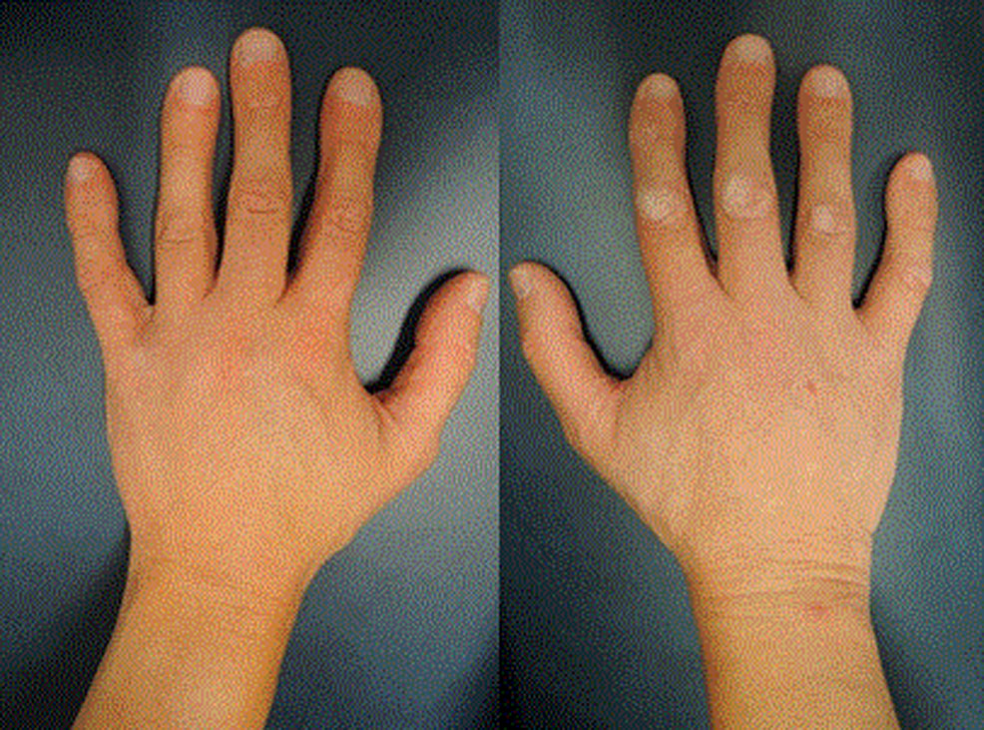
Hands at age 34. Note: relatively small hands, with small though normally shaped nails, normal thumbs

At age 34, he was referred to our hospital for a second opinion regarding his osteoporosis and non-union of a tibia fracture. Physical examination showed a slender man with a short stature (height 167 cm, (−2.1 SDS), with a saddle nose, absence of eyelashes and eyebrows and facial poikiloderma, (Fig. 1). He had sparse hair on the scalp, with two spots of alopecia areata and multiple small hyperpigmented macules on the trunk and arms. He had small but normal hands (Fig. 2); at the right foot, a partial 2–3 syndactyly of the right foot was observed.

His 5 years older brother has a milder phenotype, with a similar physical appearance and a mild intellectual disability. He was diagnosed with osteopenia and recurrent fragility fractures with non-union, but not with celiac disease (Table 1). Their parents were non-consanguineous.

**Table 1:** Clinical features in our patient and his brother compared with the frequencies of these features among previously reported Rothmund-Thomson patients

	Our patient	Brother of the patient	Reported frequency of RTS features^a^
Skin			
Poikiloderma	+	+	All
Hyperpigmentation	+	+	+
Hypopigmentation	+	+	+
Calcinosis cutis	+	−	Uncommon
Osteoma cutis	+	−	−
Palmoplantar hyperkeratosis	−	−	30%
Photosensitivity	−	−	+/−
Hair			50%
Sparse hair	+	+	
Absent eyelashes	+	+	
Sparse/absent eyebrows	+	+	
Alopecia areata	+	−	
Dental abnormalities	+	−	27–59%
Growth			
Low birth weight	+	+	+
Short stature	+	+	+
Skeleton			68–75%
Radial ray defects	−	−	20%
Metaphyseal changes	+	−	+
Osteopenia/osteoporosis	+	+	+
Small patellae	+	−	+
Ocular lesions			
Cataract	−	−	10–50%
Gastrointestinal features			17%
Oesophageal or pyloric stenosis	+	−	
Feeding problems	+	−	
Chronic emesis/diarrhoea	+	−	
Hematologic abnormalities	Hodgkin’s lymphoma	−	Occasionally
Cancer			
Osteosarcoma	−	−	30%
Skin cancer	−	−	5%
Lymphoma	+	−	Two cases^b^
Neurocognitive development			No specific data available
Mild intellectual disability	+	+	
Cytogenetic abnormalities	Mosaic trisomy 8 (15%)	Mosaic trisomy 8 (13%)	Cases reported with Mosaic trisomy 2, 7 or 8
	Mosaic isochromosome 8q (13%)^c^	Mosaic isochromosome 8q (9%)^c^	Mosaic isochromosome 2, 7 or 8
*RECQL4* gene mutations	+	+	66%

^a^Frequencies derived from [2, 8, 16, 17]

^b^Cases reported by Siitonen 2009 and Simon 2010 [13, 14]

^c^Based on interphase FISH (2 probes: LSI MYC, 8q24) on 400 lymphocytes nuclei in blood

Altogether, this presentation was compatible with a clinical diagnosis of Rothmund-Thomson syndrome (RTS, OMIM 268400).

Cytogenetic testing at the age of 13 showed a normal male karyotype in a total of 50 analysed nuclei without signs of chromosomal instability, while a CytoScan HD Array (Affymetrix) at the age of 34 showed a slight excess of chromosome 8q, suggestive for a mosaic chromosome 8q duplication (presumably between 12 and 18%). Subsequently, karyotyping and FISH analysis were performed on cultured lymphocytes from both brothers, showing a mosaicism for trisomy 8, isochromosome 8q and a normal karyotype (Table 1). Sanger sequencing of the *RECQL4* gene (OMIM 603780) showed two compound heterozygous recurrent pathogenic mutations in both brothers: one frame shift mutation: c.1048_1049delAG (p.(Arg350fsX21)) and one splice site mutation c.1391-1G>A (p.(?)). Carrier testing in the parents confirmed biallelic inheritance. Altogether, these findings confirmed the clinical diagnosis of RTS in both brothers.

One year later, the index patient was diagnosed with a stage I Hodgkin’s lymphoma in the neck for which he was started on three cycles of adriamycin, bleomycin, vinblastin, darcabazin and prednisone in combination with involved node radiation with 12 × 1.8 Gy resulting in a complete remission. He developed fever and neutropenia after the first chemotherapy. The neutropenia has resolved after a temporary break of chemotherapy and treatment with antibiotics. Neutropenia did not return after continuation of the ABVD therapy. The radiotherapy did not lead to considerable side effects.

## Discussion

In this report, we presented a case of RTS alternatively diagnosed as COPS. RTS is a rare autosomal recessive genodermatosis with a distinctive phenotype, characterised by poikiloderma, sparse hair, skeletal abnormalities and an increased risk for osteosarcoma [8]. RTS had been considered, but was assumed less likely, due to the absence of cataract and photophobia, a major sign, and the presence of osteoma cutis, whereas genetic confirmation was not possible in that period [11]. However re-evaluation of the patient and his brother changed the diagnosis to RTS and later the cataract was not associated with *RECQL4* mutations anymore.

The absence of the cataract makes the distinction between the two clinical variants of RTS: the form with poikiloderma and ocular defects, named RTSI, and poikiloderma, skeleton defects, predisposition to cancer and *RECQL4* mutations, named RTSII, which accounts for approximately 66% of RTS patients (Table 1). Osteoma cutis has not been described before; calcinosis cutis has been linked to RTS [1, 4].

The *RECQL4* gene on the long arm of chromosome 8 (8q24.3) codes for an ATP-dependent DNA helicase, which plays a role in regulating DNA replication, DNA repair and chromosomal integrity [6, 13]. *RECQL4*-deficient mice show abnormal karyotypes and aneuploidy [10], as well as defects in osteoblast progenitors [18]. In patients with RTS, these defects in osteoblast progenitors make them prone to osteosarcoma and low-turnover osteoporosis with a predisposition for fracture non-union [9, 18]. Chromosomal abnormalities, like the mosaic trisomy 8 and i(8q) have been reported in RTS patients [7]. The specific *RECQL4* mutations, c.1048_1049delAG in exon 5 and c.1391-1G>A in intron 7, were both previously reported in patients with RTS [3, 8]. One patient was reported with the exact combination of mutations; contrary to our patient, this patient showed additional humoral immune deficiency and granulomatous skin lesions [3].

Generally, intellectual disability is not considered to be a feature of RTS [8, 13, 17]. As a consequence, the number of RTS patients with a mild intellectual disability, like our patients, may be underestimated. Mild to moderate intellectual disability has been reported in a small number of cases [5, 8, 15, 16]. Co-occurring features like hydrocephalus and craniosynostosis may have played a role in the ID [16], and in some cases the diagnosis of RTS was not confirmed [5]. In our case, the history of meningitis may have attributed to some part of the intellectual disability; however, the brother was affected as well, implying a relationship with RTS. We hypothesised that the mosaic chromosomal abnormalities observed are responsible for the development of intellectual disability. However, the chromosomal imbalances were apparently acquired. Additionally, mutations in RecQ DNA helicase genes may mildly impact intellectual development, such as observed in Bloom syndrome (OMIM 210900), a chromosomal breakage syndrome, caused by mutations in the *RECQL3* gene (OMIM 604610). Although most affected individuals with Bloom syndrome have normal intellectual ability, many exhibit learning disability [12]. Likewise, a relative mild intellectual disability, such as in our patients, may be underreported in RTS patients and considered to be normal variability of intelligence. Modifier genes and co-morbidity may also play a role in the variability and atypical expression with or without ID; these may explain the phenotypic differences between the brothers.

In conclusion, we reclassify a patient with COPS as RTS with osteoma cutis and a mild intellectual disability, refuting COPS as a separate entity, since there were no reports in literature after 1991. To link mild intellectual disability to RTS, more studies are needed.

## Abbreviations

BMD: Bone marrow density
COPS syndrome: Calcinosis cutis, osteoma cutis, poikiloderma and skeletal abnormalities
DNA: Deoxyribonucleic acid
FISH: Fluorescent in situ hybridisation
ID: Intellectual disability
LSI MYC: Locus-specific identifier (LSI) MYC, 2 FISH probes used to visualise locus 8q24 on chromosome 8
RECQL4: DNA helicase, RECQ-like, type 4
RTS: Rothmund-Thomson syndrome
